# Deep learning on electrocardiogram waveforms to stratify risk of obstructive stable coronary artery disease

**DOI:** 10.1093/ehjdh/ztaf020

**Published:** 2025-03-18

**Authors:** Rishi K Trivedi, I Min Chiu, John Weston Hughes, Albert J Rogers, David Ouyang

**Affiliations:** Department of Cardiology, Cedars-Sinai Medical Center, Smidt Heart Institute, 127 S San Vicente Boulevard #A3600, Los Angeles, CA, USA; Department of Cardiology, Cedars-Sinai Medical Center, Smidt Heart Institute, 127 S San Vicente Boulevard #A3600, Los Angeles, CA, USA; Division of Cardiology, Department of Medicine, Stanford University, Palo Alto, CA, USA; Division of Cardiology, Department of Medicine, Stanford University, Palo Alto, CA, USA; Department of Cardiology, Cedars-Sinai Medical Center, Smidt Heart Institute, 127 S San Vicente Boulevard #A3600, Los Angeles, CA, USA

**Keywords:** Deep learning, Artificial intelligence, Coronary artery disease, Coronary angiography, Chronic coronary disease

## Abstract

**Aims:**

Coronary artery disease (CAD) incidence continues to rise with an increasing burden of chronic coronary disease (CCD). Current probability-based risk assessment for obstructive CAD (oCAD) lacks sufficient diagnostic accuracy. We aimed to develop and validate a deep learning (DL) algorithm utilizing electrocardiogram (ECG) waveforms and clinical features to predict oCAD in patients with suspected CCD.

**Methods and results:**

The study includes subjects undergoing invasive angiography for evaluation of CCD over a 4-year period at a quaternary care centre. oCAD was defined as performance of percutaneous coronary intervention (PCI) based on assessment by interventional cardiologists during elective angiography. DL models were developed for ECG waveforms alone (DL-ECG), clinical features from standard risk scores (DL-Clinical), and the combination of ECG waveforms and clinical features (DL-MM); a commonly used pre-test probability estimation tool from the CAD Consortium study was used for comparison (CAD2) [3]. The CAD2 model [AUC 0.733 (0.717–0.750)] had similar performance as the DL-Clinical model [AUC 0.762 (0.746–0.778)]. The DL-ECG model [AUC 0.741 (0.726–0.758)] had similar performance as both the clinical feature models. The DL-MM model [AUC 0.807 (0.793–0.822)] had a superior performance. Validation in an external cohort demonstrated similar performance in the DL-MM [AUC 0.716 (0.707–0.726)] and CAD2 risk score [AUC 0.715 (0.705–0.724)].

**Conclusion:**

A multi-modality DL model utilizing ECG waveforms and clinical risk factors can improve prediction of oCAD in CCD compared with risk-factor based models. Prospective research is warranted to determine whether incorporating DL methods in ECG analysis improves diagnosis of oCAD and outcomes in CCD.

## Graphical Abstract

**Graphical Abstract:**
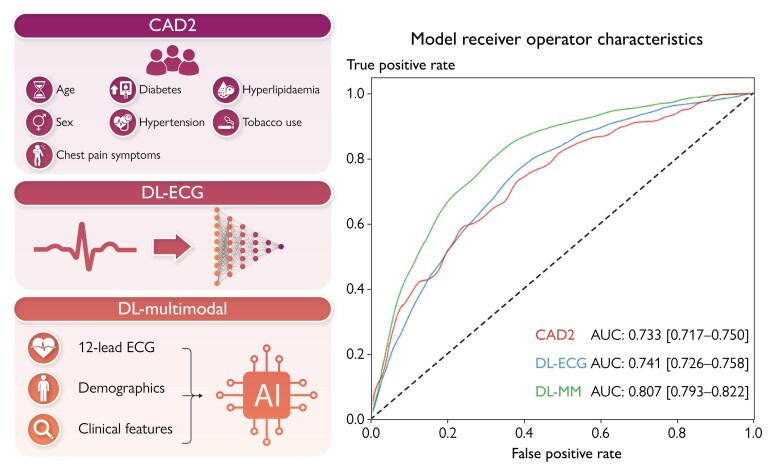

## Introduction

Cardiovascular disease is the leading cause of death in the United States. Cardiovascular mortality is driven largely by coronary artery disease (CAD). The prevalence of CAD is on the rise with an estimated 20.1 million adults in the United States being affected.^1^ CAD causes a variety of disease states ranging from acute coronary syndromes (ACS) such as myocardial infarction through chronic coronary diseases (CCD) such as angina pectoris. Over the past few decades, the prevalence of ACS has been stable while the proportion of individuals with CAD who have CCD has risen.^1–3^ According to the most recent data from the American Heart Association (AHA) and the American College of Cardiology (ACC), the prevalence of CCD is 11 million comprising the majority of the 20.1 million adults in the United States with CAD related diseases.^4^ Despite the immense burden on human health, current tools for CAD risk stratification are insufficient.

The AHA and ACC recommend the use of pre-test probability (PTP) risk assessment to stratify patients suspected to have CAD.^5^ Based on PTP, patients are assessed a risk score to determine whether they need further evaluation for obstructive CAD (oCAD) and what type of testing to utilize. PTP scores such as the Updated Diamond-Forrester Score,^6^ the Duke Clinical Score,^7^ the CAD Consortium Score (CAD2),^8^ and the ACC/AHA-PTP^9^ are derived from large patient cohorts and weigh history-based factors such as demographics, co-morbidities, social risk factors, and description of chest pain. Unfortunately, these risk scores overestimate CAD prevalence leading to over-testing.^10^ The ACC/AHA-PTP has been shown to overestimate CAD prevalence by a factor of 2.6.^9^ At the same time, PTP tools under-estimate CAD risk in women with lower utilization of additional CAD evaluation.^11,12^ Similar under-appreciation of CAD risk has been seen in minorities including African-Americans,^13,14^ Hispanics,^15^ and South Asians.^16,17^ A large-scale study of 50 561 patients with CAD symptoms who underwent anatomic evaluation for CAD showed that guideline-recommended PTP risk estimation has only a moderate area under the receiver-operator curve performance of 0.715.^9^ Accurate CAD risk assessment is important to allow early initiation of therapies that are known to improve outcomes.

Electrocardiograms (ECGs) are a widely available, non-invasive, low-cost, low-risk, and rapid point-of-care cardiovascular test that can detect a wide variety of cardiovascular disorders. ECGs are frequently obtained for patients suspected of having CAD or with known CCD. While there are ECG changes that show active ischaemia and features suggestive of existing myocardial damage, there are no ECG changes that reliably demonstrate obstructive CAD without active ischaemia or undiagnosed CAD. Although there are no features conventionally recognized, the vast information contained in ECGs is under-appreciated due to limitations present in human perception.

Artificial intelligence (AI) including deep learning (DL) can be used to overcome this limitation in human perception. DL has successfully been applied to ECG interpretation of a variety of cardiac conditions including CAD.^18^ Some of the early work in this space was focused on using DL to improve ECG interpretation of ACS to streamline diagnosis of conventional ECG definitions of ACS as well as identifying new patterns that were previously under-appreciated.^19^ As the field broadens, DL-based ECG analysis has also shown power in identifying sub-clinical CAD such as that identified by coronary artery calcification.^20,21^ There are also applications of DL to ECG analysis to identify disease states previously not thought to not have classic ECG features such as the development of atrial fibrillation when in normal sinus rhythm.^22^ DL on ECG waveforms has also been shown to hold prognostic utility to determine future risk of atherosclerotic cardiovascular diseases including CAD.^23^

The ability of DL to stratify an individual’s risk of oCAD has not been fully evaluated but has important implications in the detection and risk stratification for individuals with concern for CAD. The present study was designed to develop and evaluate whether a DL model trained on ECG data can improve discrimination of oCAD in subjects with suspected CCD compared with conventional risk scores using performance of percutaneous coronary intervention (PCI) as a surrogate marker of oCAD.

## Methods

### Study population

Patients aged 18 years or older referred to Cedars-Sinai Medical Center (CSMC) for coronary angiography between 1 April 2018 and 15 April 2022 with at least one 12-lead ECG performed within the five years prior to their angiography were included in the study. Subjects suspected of having ACS (non-ST-elevation, ST-elevation myocardial infarction, or unstable angina) at the time of angiography as defined by International Classification of Diseases (ICD) code were excluded. This yielded a population at risk for CCD—predominantly including subjects with concern for CCD and a smaller subset of patients undergoing angiography for pre-operative risk stratification, evaluation of aetiology of new onset cardiomyopathy, or prior to valvular heart disease intervention at the time of evaluation. This resulted in 8361 angiography studies performed which were matched with 40 602 ECGs. The ECGs were randomly divided in an 8:1:1 ratio at the patient level for model development, validation, and testing, respectively. The primary aim of the study was to predict the presence of oCAD using whether PCI was performed as a surrogate marker. For external validation, we identified 71 755 ECGs from 24 533 coronary angiograms performed in 17 012 patients at Stanford HealthCare (SHC) from August 2005 to May 2018 with the same exclusion criteria as the CSMC cohort. This study was approved by the institutional review boards of both CSMC and SHC. Due to the retrospective nature of the study using de-identified ECG and electronic health record data, patients’ informed consent was waived.

### Data collection

ECGs from CSMC were recorded using a GE machine, following the standard 10-second, 12-lead ECG protocol. These ECG waveforms were captured at a sampling rate of 500 Hz and represented as 10-second matrices of 12 × 5000 amplitude values. For external validation at SHC, ECGs were recorded using the Philips TraceMaster system, also at a 500 Hz sampling rate and formatted as 12 × 5000 matrices. Clinical features and demographic information such as age, sex, race, and medical history were collected from the electronic medical record at the respective institutions. Clinical information was obtained from the structured database of the ACC National Cardiovascular Data Registry and using ICD codes.

### Model training

Three DL models were developed using neural network frameworks to predict oCAD: using ECG waveform data alone (DL-ECG), using clinical features alone (DL-Clinical), and both ECG waveform and clinical features (DL-MM). In addition to the DL models, we calculated PTP risk score using the CAD2 score to use as a comparator. CAD2 was selected given its superior performance amongst available PTP risk estimation tools in real-world practice and its global rather than regional use.^6,24–26^

The DL-ECG model was trained using a convolutional neural network (CNN) tailored for ECG interpretation developed with the PyTorch DL framework (see Supplementary material online, *Figure S1*). Initially, the model started with randomly assigned weights and was trained using a binary cross-entropy loss function for up to 100 epochs. We used an ADAM optimizer with an initial learning rate of 0.01. Training included an early stopping mechanism, which halted the process based on the area under the receiver operating characteristic curve (AUC) from the validation dataset comparing the predicted probability to whether PCI was performed.

The DL-Clinical model was trained using a 3-layer feedforward neural network (FNN) with 32, 64, and 32 neurons in each layer, respectively. The input variables for this mode were the same clinical characteristics used to calculate the CAD2 risk score. The same training hyperparameters were used in development as in the DL-ECG CNN model.

The DL-MM is a multi-modal model using the same FNN structure as the clinical characteristic mode with addition of the predictive output from the ECG-based CNN model to the clinical data for training.

### Statistical analysis

Analyses were conducted on the held-out test dataset comprising 10% of the CSMC cohort, which was not used during model training, and the external cohort from SHC.

Statistical analysis was performed in Python. We reported continuous variables using the median and inter-quartile range (IQR) and categorical variables using the number and percentage. We assessed the models’ performance in predicting the primary outcome using performance characteristics including AUC, sensitivity, specificity, positive predictive value, negative predictive value, and calibration plots. We computed two-sided 95% confidence intervals using 1000 bootstrapped samples for each calculation to compare performance between models.

A Shapley additive explanations (SHAP) analysis was performed on the combination model to determine which of the included features had the largest contribution to model output in the multi-modal model.

## Results

### Study population

**Figure 1:**
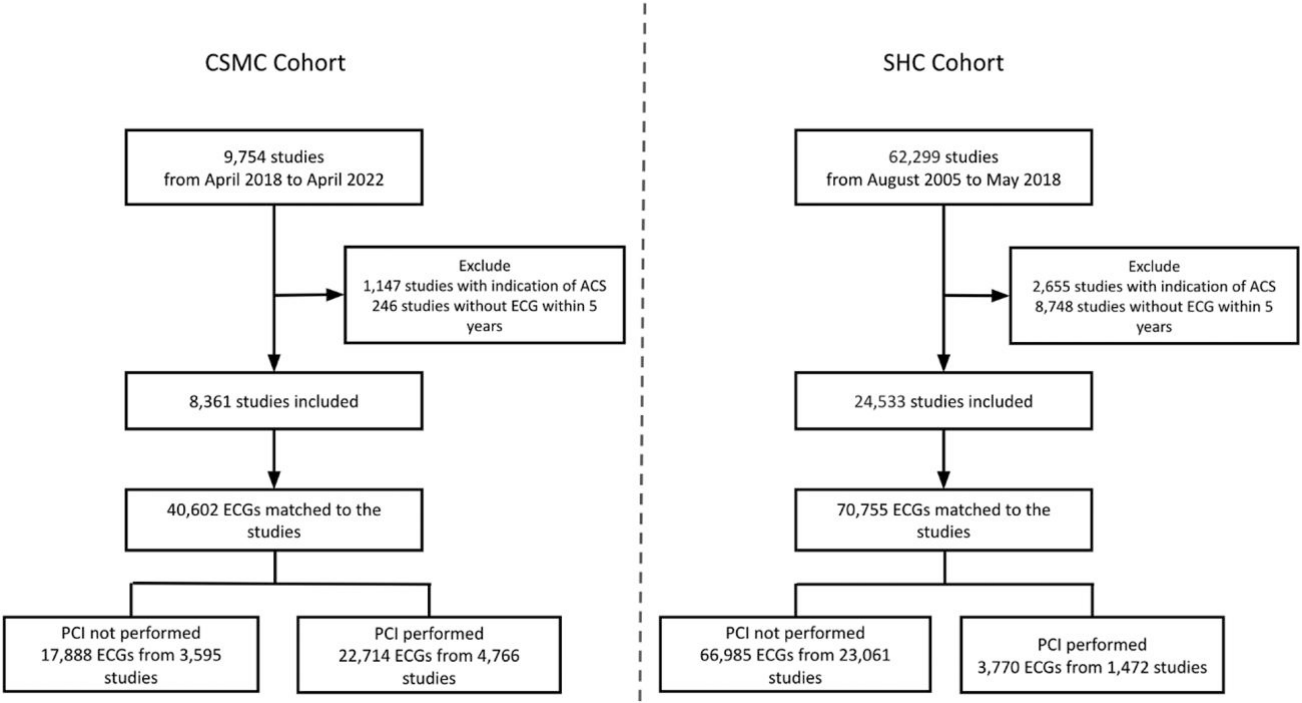
Consort diagram. Consort diagram depicting the internal and external validation cohorts. Exclusions were made for subjects where indication for coronary angiography was an acute coronary syndrome and if the subject did not have an ECG within 5 years of coronary angiography. These studies were paired with all ECGs obtained within 5 years of coronary angiography. Label for presence of obstructive coronary artery disease was whether percutaneous coronary intervention was performed.

Between 1 April 2008 and 15 April 2022, we identified 9754 coronary angiograms at CSMC. After exclusions, 8361 studies remained which were matched to a total of 40 602 ECGs within 5 years of the studies. Between 1 August 2005 and 31 May 2018, we identified 62 299 coronary angiograms at SHC. After exclusions, 24 533 coronary angiograms remained, which were matched to a total of 70 755 ECGs within 5 years of the studies (*Figure 1*).

In the development set from CSMC, there were 36 542 total ECGs. The median age was 67 years (IQR 57–76 years), 71.6% of the patients were male, and the majority were White (48.3%). Medical co-morbidities include hypertension (36.7%), diabetes mellitus (31.5%), dyslipidaemia (40.0%), and smoking (7.6%). Baseline ECG abnormalities were notable for right bundle branch block (RBBB) (9.9%), left ventricular hypertrophy (LVH) (5.9%), and left bundle branch block (LBBB) (5.2%) (*Table 1*).

**Table 1:** Cohort characteristics

	Cedars Sinai Medical Center		Stanford Healthcare
	Development set	Hold-out test set	External test set
Total ECGs	36 542	4060	70 755
	Median (IQR)/N (%)	Median (IQR)/N (%)	
Age	67 (57–76)	62 (53–70)	60 (52–68)
Male sex	26 177 (71.6%)	3021 (74.4%)	42 807 (60.6%)
Race			
White	17 638 (48.3%)	1242 (30.6%)	
Black	2979 (8.2%)	192 (4.7%)	
Hispanic	1913 (5.2%)	134 (3.3%)	
Asian	1980 (5.4%)	234 (5.8%)	
Others	12 032 (32.9%)	2258 (55.6%)	
Medical history			
Hypertension	13 396 (36.7%)	754 (18.6%)	38 420 (54.3%)
Diabetes mellitus	11 496 (31.5%)	936 (23.1%)	18 184 (25.7%)
Dyslipidaemia	14 605 (40.0%)	1230 (30.3%)	33 679 (47.6%)
Smoker	2793 (7.6%)	134 (3.3%)	
ECG			
Sinus rhythm	23 260 (63.7%)	1521 (37.5%)	51 793 (73.2%)
Atrial fibrillation/flutter	3003 (8.2%)	224 (5.5%)	
QT prolongation	18 381 (50.3%)	1141 (28.1%)	
Left ventricular hypertrophy	2148 (5.9%)	148 (3.6%)	7783 (11.0%)
Left bundle branch block	1883 (5.2%)	74 (1.8%)	3325 (4.7%)
Right bundle branch block	3607 (9.9%)	263 (6.5%)	8915 (12.6%)
Percutaneous coronary intervention	21 210 (58.0%)	1504 (37.0%)	3770 (5.3%)

Characteristics of the internal and external cohorts. Displayed characteristics are either shown as median and inter-quartile range or number with percent of cohort with the listed feature. There are differences amongst the internal validation and test cohorts, which are due to random segmentation of the cohort; these differences include distribution of ethnicity, prevalence of co-morbidities, and baseline ECG abnormalities. Compared with the Cedars Sinai Medical Center Cohort, the external test set from Stanford Healthcare has fewer males, differences in co-morbidities, prevalence of baseline ECG changes, and the proportion of individuals who received a percutaneous coronary intervention.

IQR, inter-quartile range.

In the hold-out test set at CSMC, 4060 ECGs were analysed with PCI performed in 1504 cases (37.0%). The median age was 62 years (IQR 53–70), 74.4% were male, and the majority did not identify as White/Black/Hispanic/Asian (55.6%). Co-morbidity prevalence included hypertension (18.6%), diabetes mellitus (23.1%), dyslipidaemia (30.3%), and smoking (3.3%). Baseline ECG abnormalities was notable for RBBB in 6.5%, LVH in 3.6%, and LBBB in 1.8% (*Table 1*).

In external validation within the SHC cohort, 70 755 ECGs were analysed with PCI performed in 3770 cases (5.3%). The median age in the SHC cohort was 60 years (IQR 52–68) and 60.6% were male, ethnicity was not reported. Co-morbidity prevalence included hypertension (54.3%), diabetes mellitus (25.7%), and dyslipidaemia (47.6%). Baseline ECG abnormalities was notable for RBBB (12.6%), LVH (11.0%), and LBBB (4.7%) (*Table 1*).

### Model performance in the primary cohort

**Figure 2:**
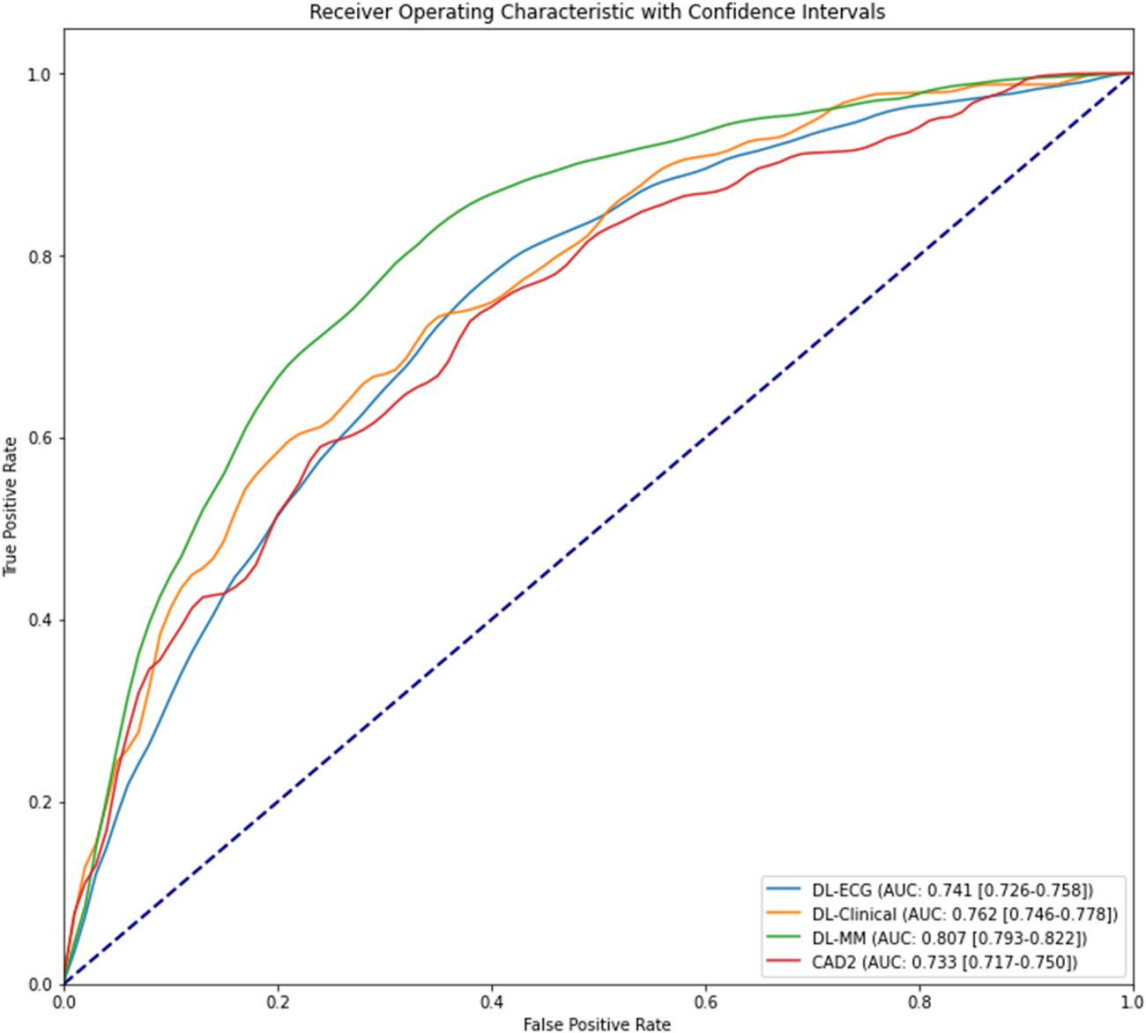
AUC of model performance. Visual representation of the area under the receiver-operator curve characteristics including all developed models. The deep learning multi-modal model had a visually distinct performance curve consistent with its statistically significant superior performance as seen in *Table 2*. AUC, area under the receiver-operator curve; CAD2, coronary artery disease consortium risk estimation tool; DL-clinical, deep learning model trained on clinical features alone; DL-ECG, deep learning model trained on ECG waveforms only; DL-MM, deep learning model trained on ECG waveforms and clinical features.

The DL-ECG model, trained on ECG waveform data alone, achieved an AUC of 0.741 (95% CI: 0.726–0.758) in predicting oCAD as defined by need for PCI in the test cohort from CSMC. The DL-clinical model, trained on clinical features alone, achieved an AUC of 0.762 (95% CI: 0.746–0.778). The DL-MM model, the multi-modal approach that integrated both ECG and clinical data, achieved an AUC of 0.807 (95% CI: 0.793–0.822). The traditional CAD2 clinical model, initially developed with logistic regression on a large patient cohort, resulted in an AUC of 0.733 (95% CI: 0.717–0.750). Despite having no imputed clinical variables, the DL-ECG model had equivalent performance characteristics as clinical feature-based models. The multi-modality model improved performance characteristics beyond that achieved by clinical features alone by incorporation of ECG features (*Table 2* and *Figure 2*). The calibration plot demonstrates that DL-MM has superior calibration characteristics compared with DL-ECG (see Supplementary material online, *Figure S2*). Additional information regarding the specifics regarding impact of model output cut-offs on sensitivity and specificity is provided in the supplement (see Supplementary material online, *Table S1*).

**Table 2:** Model performance characteristics

	Hold-out test set	External validation
	AUC (95% confidence interval)	AUC (95% confidence interval)
CAD2	0.733 (0.717–0.750)	0.715 (0.705–0.724)
DL-ECG	0.741 (0.726–0.758)	0.633 (0.623–0.642)
DL-Clinical	0.762 (0.746–0.778)	
DL-MM	0.807 (0.793–0.822)^a^	0.716 (0.707–0.726)

Performance characteristics of the developed models in the internal hold-out test set and in the external validation cohort. Results are shown as area under the receiver-operator curve with 95% confidence intervals. In the internal test cohort, model performance was lowest by CAD2 but roughly similar to that achieved by a deep learning model trained only on ECGs and a deep learning model trained only on clinical features; the multi-modal model had a statistically superior performance than the other models. In the external validation group, the CAD2 model and multi-modal deep learning model had similar performance with no statistically significant differences.

^a^Statistically significant differences defined by *P*-value < 0.05 compared with the CAD2 model.

AUC, area under the receiver-operator curve; CAD2, coronary artery disease consortium risk estimation tool; DL-clinical, deep learning model trained on clinical features alone; DL-ECG, deep learning model trained on ECG waveforms only; DL-MM, deep learning model trained on ECG waveforms and clinical features.

**Figure 3:**
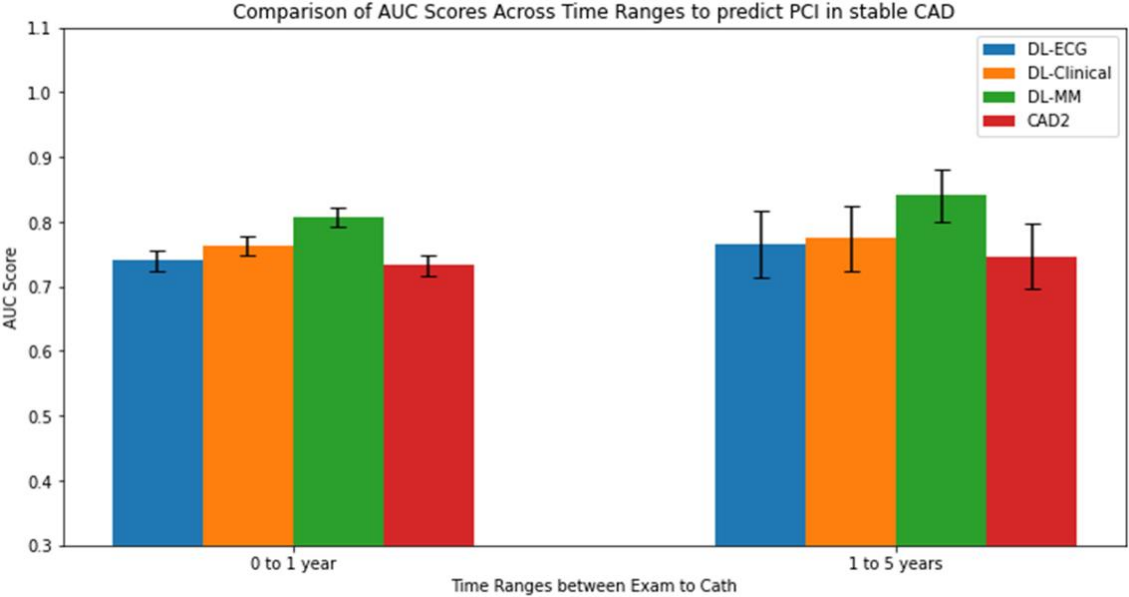
Model performance across time for detection of obstructive coronary artery disease. Changes in model performance based on timing of ECG prior to coronary angiogram. The deep learning multi-modal model had superior performance compared with the CAD2 model, which was preserved regardless of whether the ECG was obtained within 1 year of the angiogram or from 1 to 5 years prior to angiography. AUC, area under the receiver-operator curve; CAD2, coronary artery disease consortium risk estimation tool; DL-clinical, deep learning model trained on clinical features alone; DL-ECG, deep learning model trained on ECG waveforms only; DL-MM, deep learning model trained on ECG waveforms and clinical features; PCI, percutaneous coronary intervention.

When analysing model performance from ECG to catheterization over different time periods, the multi-modal model consistently outperformed both the ECG-only and CAD2-only models. It achieved an AUC of 0.806 (95% CI: 0.791–0.821) from 0 to 1 year and an AUC of 0.841 (95% CI: 0.801–0.880) from 1 to 5 years. Using the CAD2 clinical score, predictability for PCI was also lower, with an AUC of 0.734 (95% CI: 0.716–0.749) for 0 to 1 year and an AUC of 0.746 (95% CI: 0.696–0.796) for 1 to 5 years (*Figure 3).*

### Subgroup analysis

**Figure 4:**
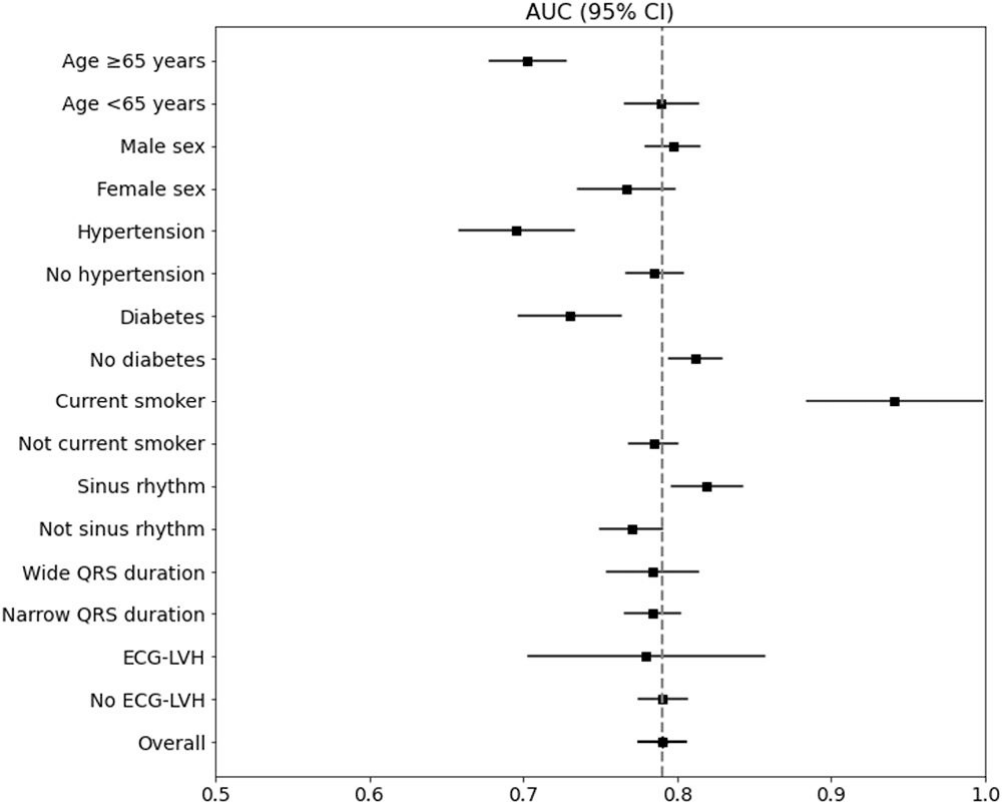
Multi-modal model performance stratified by subgroup. Forest plot depicting performance of the multi-modal model stratified by subgroups. The deep learning multi-modal model had mostly consistent performance regardless of subgroup. The model had slightly reduced performance in younger subjects, hypertensive patients, and diabetic patients; the model had slightly higher performance in current smokers. AUC, area under the receiver-operator curve; CAD2, coronary artery disease consortium risk estimation tool; CI, confidence interval; LVH, left ventricular hypertrophy.

The DL-MM model’s predictive performance was stratified across various patient demographics, clinical characteristics, and ECG rhythms. The model was affected by demographics, showing lower performance in patients over 65 years old with an AUC of 0.725 (95% CI: 0.701–0.748). The model performed slightly worse in hypertensives with an AUC of 0.690 (95% CI: 0.652–0.727) and in diabetics with an AUC of 0.766 (95% CI: 0.739–0.794). The model performed significantly better in smokers with an AUC of 0.961 (95% CI: 0.914–1.000). The model’s predictive performance was largely consistent across subgroups including non-sinus rhythm, wide QRS complexes, or patterns of LVH, with notably superior performance in sinus rhythm with an AUC of 0.833 (95% CI: 0.811–0.855) (*Figure 4).* Subgroup analysis for the CAD2 model is shown in the supplemental section (see Supplementary material online, *Figure S3).*

### Model explainability

**Figure 5:**
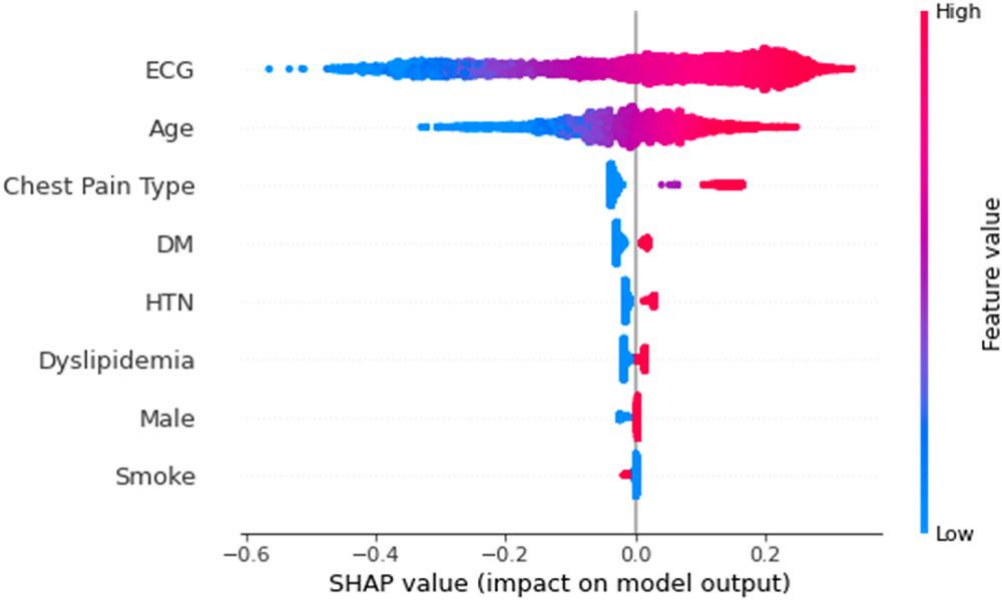
Shapley additive explanations (SHAP) analysis of multi-modal model. SHAP analysis of the multi-modal model depicting, which features included in the multi-modal model had the most impact on model output. The main feature that impacted multi-modal model output was ECG waveform analysis followed by age and chest pain type with limited effect on model output based on the remaining clinical variables. DM, diabetes mellitus; HTN, hypertension; SHAP, Shapley additive explanations.

We utilized SHAP algorithms to generate a ranking of features that explain the output of our multi-modal model. The SHAP analysis depicts the impact of different features on model output wherein the features that have most significant positive or negative impact on model output are seen with having more spread along the X-axis with the colour depicting the feature importance as high or low. The most influential feature on the model’s classification output was the ECG waveform analysis, followed by age and type of chest pain with little contribution based on the subject’s underlying diagnoses. According to the SHAP values, older patients, those with typical chest pain, diabetes mellitus, and hypertension were more likely to receive a positive prediction for PCI. Conversely, patients who were female or had a history of smoking were less likely to receive a positive prediction *(Figure 5)*.

### Model performance in the external validation cohort

In the SHC cohort, the DL-ECG model achieved an AUC of 0.633 (95% CI: 0.623–0.642) in predicting oCAD as defined by need for PCI. The multi-modal approach, DL-MM showed a superior performance with an AUC of 0.716 (95% CI: 0.707–0.726). The CAD2 clinical model using published weights achieved an AUC of 0.715 (95% CI: 0.705–0.724) in predicting oCAD as defined by need for PCI (*Table 2*).

## Discussion

In this study, we demonstrated that DL on ECG waveforms can be used to risk stratify patients with concern for CCD in having oCAD as defined by need for PCI. This includes the ability of DL on ECG waveform to stratify patients with similar accuracy as clinical variable-based models even without incorporation of clinical features. Further, we showed that in our study population, DL on ECG waveforms can improve risk stratification of oCAD beyond that afforded by currently recommended PTP risk estimation. The performance characteristics diminished in external validation wherein a multi-modal model including DL on ECG waveforms performed similarly to PTP risk estimation. This is the first study to show that DL on ECG waveforms holds utility in the diagnosis of oCAD as defined by need for PCI in subjects with concern for CCD and builds on the use of DL on ECG waveforms to improve CCD diagnosis and risk stratification.

Recently, significant work has been done in the application of DL to ECG waveforms including work on improving ECG interpretation for CAD. This work has been focused on improving interpretation of ECG waveforms in ACS to better identify individual’s whose ECG is consistent with occlusive myocardial infarction including those outside the conventional ECG criteria for ST-elevation myocardial infarction.^27,28^ This has led to the development and now implementation of these algorithms within care delivery systems and at point of care.^29^ However, unlike in ACS where there are known ECG features that are being standardized using DL, the present study suggests that there are additional ECG features that are not appreciated by clinicians that represent pathologic changes in coronary arteries without the development of active ischaemia. Our group has previously demonstrated similar occult ECG changes that are consistent with sub-clinical disease such as future development of atrial fibrillation from an ECG in normal sinus rhythm or the presence of left ventricular systolic dysfunction.^22,30^ Previous work on the field of CCD has shown promise with studies demonstrating that DL on ECG waveforms can be used for alternative methods of CAD diagnosis such as the degree of coronary artery calcification as defined by coronary artery calcium score^20^ and for non-obstructive or sub-clinical CAD.^21^

Unfortunately, with current computer science methods we are not able to interrogate our algorithm to determine specifically which ECG waveform changes are being used in prediction. Tools such as SHAP analysis allow us to glean information regarding the impact of model features whereby we can identify that ECG waveform analysis is the primary feature providing discriminatory power. Additional explainability analysis tools with gradient based methods such as saliency maps and Grad-CAM can also identify what ECG components are being highlighted in model output but were unrevealing in our study.^31,32^ This limitation of DL explainability analysis has been a barrier for clinical implementation but does not detract from the successful prediction characteristics. As machine learning tools and analysis techniques develop over time, hopefully this limitation will decrease, and we can reverse engineer to identify the hallmark ECG features the current model is using to determine its performance. In the meantime, future work on clinical applicability and safety of these tools is paramount to clinical incorporation.

This study suggests that the use of DL on ECG analysis of individuals with concern for CCD may be a feasible strategy for identifying and stratifying patients with concern for CCD. The reduced performance in an external cohort will require further evaluation and subsequent studies are needed to more comprehensively evaluate the generalizability of the findings. There are a variety of differences that contribute to the decreased performance including technical aspects such as ECG acquisition; however, population differences between the two cohorts are likely the primary contributor. As seen in *Table 1*, there was a lower proportion of males, differences in co-morbidities, higher proportion of individuals in sinus rhythm, and perhaps most importantly a significantly lower prevalence of oCAD as defined by need for PCI. Given the known performance characteristics of the model, it is unsurprising that performance would be lower in a population with lower disease prevalence. Further research is warranted into whether the application of DL to ECG in CCD can be generalized to diverse populations both in demographics and disease prevalence by investigating model performance at other centres.

Previous diagnostic evaluation of CCD relied upon large patient cohorts and logistic regression to identify the salient features that correlate with oCAD. Unfortunately, these cohorts often had poor representation of some patient subgroups including women and minorities which led to poor discrimination when applied to patients in these subgroups. In the present study, DL on ECG waveforms alone was able to discriminate the presence of oCAD to a similar degree as models in which clinical features were directly imputed. As seen in the subgroup analysis, the model performance is not significantly reduced in demographic subgroups or by specific co-morbidities. This suggests that DL models may perform superior in real-world practice where patient’s demographics do not closely match the cohorts from which PTP risk scores were derived.

PTP models also rely upon fixed characteristics of a patient rather than the current pathophysiologic state. In clinical practice, there is vast heterogeneity on the impact of co-morbidities on patient presentation; for example, in smokers, there is a breadth of disease effect ranging from early CAD to absence of significant cardiovascular complications. The use of DL on ECG waveforms allows for detection of developed pathophysiology rather than potential effects based on population data alone. This progress towards personalized evaluation is crucial in the improvement of diagnostic testing for CAD wherein there is a high amount of variability within patient presentation. DL models such as the one developed in this study could be deployed in clinical settings to better stratify a patient’s risk of CAD and guide downstream diagnostic testing. Improved risk estimation could allow more effective utilization of advanced cardiovascular testing and to triage those with highest risk to early testing and those with lower risk to more judicious evaluation with higher specificity tests. This adjustment to diagnostic evaluation of CAD is especially important in low resource settings where advanced testing is limited while ECGs are rapidly available and low-cost. Identifying those at highest risk would allow optimization of referrals for cardiac catheterization reducing wait times, reducing procedural risk, and improving cost allocation within the healthcare system.

The DL-MM model retains high performance characteristics, superior to clinical features alone, even when the ECG is evaluated 1–5 years prior to angiography. This suggests that the model may be identifying characteristics on the ECG that are related to sub-clinical atherosclerosis. This warrants further investigation as it would have significant implications on a public health level for detection of CAD. Currently, there are models based on traditional logistic regression as well as DL-based approaches for the prediction of total atherosclerotic cardiovascular disease, however, none exist for CAD specifically.

Ischaemic heart disease is undertreated in clinical practice despite the vast number of effective treatment tools available. Part of this is due to under-appreciation of the burden of CAD but also related to the high healthcare costs associated with definitive testing for CAD and the variability in adequate advanced testing modalities across the world. The currently proposed strategy utilizes DL but the baseline test that is needed is an ECG, which is widely available, low-cost, and carries virtually no procedural risk making it an attractive option for diagnostic testing.

The application of DL on ECG waveform analysis for stable CAD is a developing field but represents an attractive option for CAD diagnosis. Further research is warranted into the generalizability of the present findings and whether incorporation of DL into CAD diagnosis is feasible, safe, and can improve clinical outcomes.

## Study limitations

The present study was derived from a patient cohort that may not be fully representative of the general population in demographics and disease burden. The CSMC and SHC cohorts are from quaternary care centres with expertise in CAD and therefore there is a higher prevalence of oCAD than the general population. Additionally, there is a referral bias as individuals included were referred for angiography based on high index of suspicion of a cardiologist for the presence of oCAD. There are also differences in the demographics of the quaternary care population compared with the public which may reduce the generalizability of the findings. The study was done retrospectively and therefore, the presence of additional confounders including access to care, abnormal prior cardiovascular testing, and other clinical features are not well described. The study was performed with random split of the data and therefore, the derivation, validation, and test cohorts are not equivalent in terms of demographics or co-morbidities. Lastly, the outcome used for labelling of oCAD was whether PCI was performed, which is in part subjective based on the operator; therefore, the presence of oCAD that was not amenable to PCI or better managed with surgical revascularization is unknown in the present study. Similarly, anatomic definition of CAD has its limitations with functional ischaemia being an important aspect of CAD evaluation. Therefore, future studies using alternate labels for oCAD whether it is anatomic using discrete presence of stenosis or functional CAD by hemodynamic impact is needed to support the findings of this study.

## Conclusions

A DL model on ECG waveform for evaluation of oCAD shows reasonable discrimination for the presence of oCAD as defined by need for PCI and comparable with clinical characteristics-based models. The addition of DL on ECG waveform analysis to clinical feature-based models yields superior discrimination characteristics compared with current PTP risk scores; however, this performance is mostly reserved to the derivation cohort. The performance of this algorithm maintains predictive characteristics as far out as 5 years prior to identification of oCAD and performs well across most subgroups. These findings are encouraging; however, future work is needed to evaluate the generalizability of a DL model for ECG waveform analysis to predict oCAD in CCD in diverse populations and whether utilization can improve clinical outcomes.

## Supplementary Material

ztaf020_Supplementary_Data

## Data Availability

The data underlying this article cannot be shared publicly for the privacy of individuals that participated in the study at Cedars-Sinai Medical Center and Stanford Healthcare.
