# Supplementary material for: Deep learning on electrocardiogram waveforms to stratify risk of obstructive stable coronary artery disease
Source: Eur Heart J Digit Health. 2025 Mar 18;6(3):456–65. doi: 10.1093/ehjdh/ztaf020 (PMC12088713; doi:10.1093/ehjdh/ztaf020)
Supplement: ztaf020_Supplementary_Data [file ztaf020_supplementary_data.docx]

**Supplemental Tables and Figures**

Figure S1. Deep Learning Model Architecture and Framework.


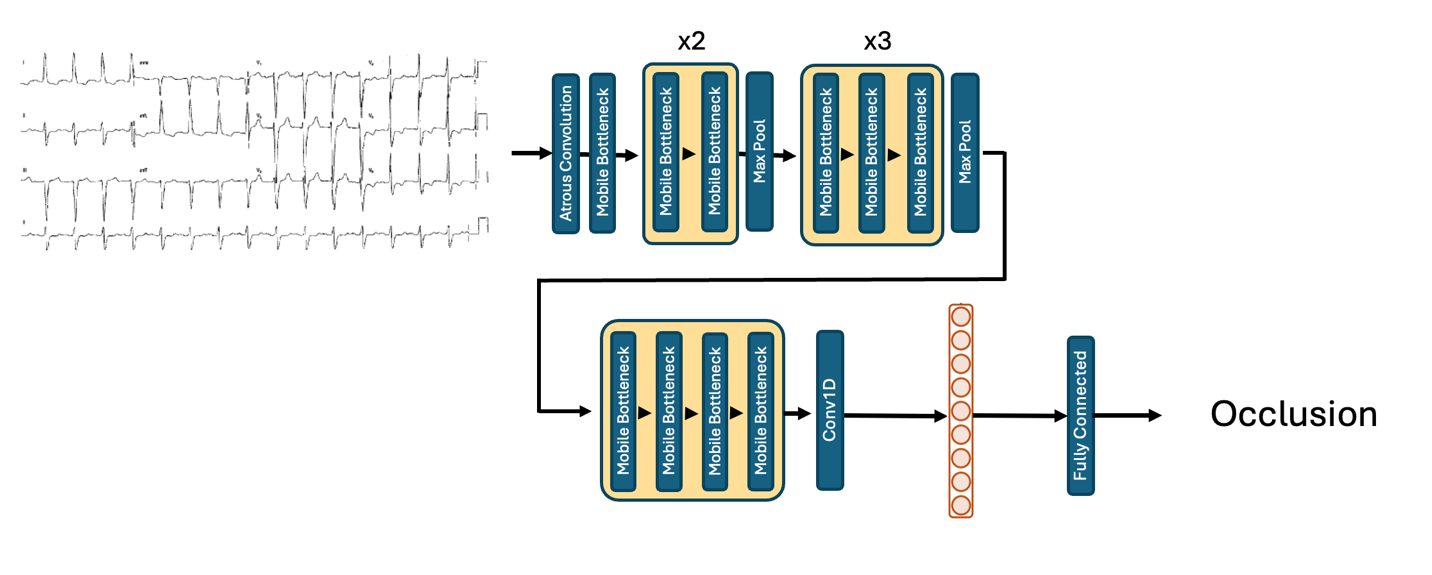


Figure S1. We employed a one-dimensional EfficientNet-based architecture to train an ECG model for predicting obstructive coronary artery disease as defined by need for percutaneous coronary intervention. The model begins with a convolution layer to capture multi-scale temporal features from the input ECG signals. This is followed by a series of mobile bottleneck layers organized into structured blocks. Specifically, the first block consists of two repeated mobile bottleneck layers, each followed by max-pooling to progressively reduce feature dimensionality while retaining critical temporal information. The second block contains three repeated mobile bottleneck layers, also interspersed with max-pooling layers. After these two blocks, the extracted features are passed through an additional set of mobile bottleneck layers for further refinement. A single Conv1D layer is applied to aggregate the temporal features into a compact, high-dimensional representation. Finally, a fully connected layer maps the learned features to a single output, predicting coronary artery occlusion. This architecture balances computational efficiency with the ability to capture intricate temporal patterns in ECG signals.

Figure S2. Calibration Plot for Deep Learning Models.


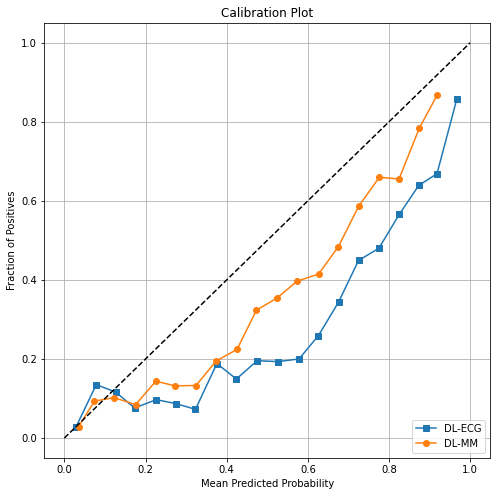


Figure S2. Calibration plot comparing the ECG based deep learning model and the multimodal deep learning model for predicting obstructive coronary artery disease as defined by need for percutaneous coronary intervention. The X-axis represents the predicted probability, while the Y-axis shows the observed fraction of positives (calibration). The diagonal line represents a perfectly calibrated model where predicted probabilities align exactly with observed outcomes. The deep learning multimodal model demonstrates closer alignment with the diagonal line, indicating better calibration compared to the deep learning model trained on ECG waveforms only. DL-ECG = deep learning model trained on ECG waveforms only, DL-MM = deep learning model trained on ECG waveforms and clinical features.

Figure S3. CAD2 Model Performance Stratified by Subgroup.
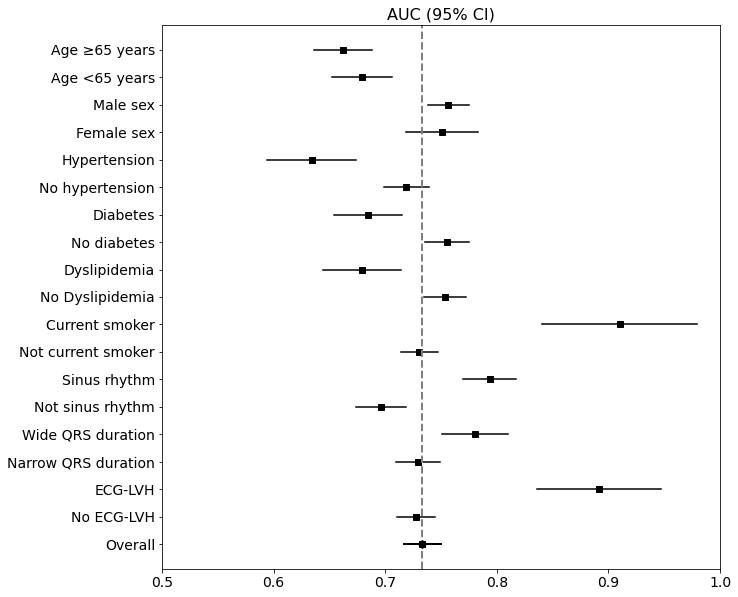


Figure S3. Forest plot depicting performance of the CAD2 model stratified by subgroups. The models' performance is slightly lower in patients with hypertension, diabetes, and dyslipidemia. The model’s performance is slightly higher in smokers and those with left ventricular hypertrophy as identified by ECG. AUC = area under the receiver operator curve, CAD2 = Coronary Artery Disease Consortium risk estimation tool, CI = confidence interval. LVH = left ventricular hypertrophy.

Table S1. Different Thresholds for Performance Metrics in the Internal Test Set.

| Threshold | Sensitivity | Specificity | PPV | NPV |
| --- | --- | --- | --- | --- |
| >0.195 | 0.950 | 0.346 | 0.461 | 0.922 |
| >0.475 | 0.845 | 0.642 | 0.581 | 0.876 |
| >0.823 | 0.299 | 0.950 | 0.777 | 0.697 |

Table S1. Performance metrics of the multimodal deep learning model based on output threshold utilized for definition of a positive result. As the cutoff increases, the sensitivity decreases with an increase in specificity. NPV = negative predictive value, PPV = positive predictive value.

**References**

1. Tsao CW, Aday AW, Almarzooq ZI, Anderson CAM, Arora P, Avery CL, et al. Heart Disease and Stroke Statistics-2023 Update: A Report From the American Heart Association. Circulation. 2023;147(8):e93-e621.

2. Benjamin EJ, Virani SS, Callaway CW, Chamberlain AM, Chang AR, Cheng S, et al. Heart Disease and Stroke Statistics-2018 Update: A Report From the American Heart Association. Circulation. 2018;137(12):e67-e492.

3. Virani SS, Alonso A, Aparicio HJ, Benjamin EJ, Bittencourt MS, Callaway CW, et al. Heart Disease and Stroke Statistics-2021 Update: A Report From the American Heart Association. Circulation. 2021;143(8):e254-e743.

4. Virani SS, Newby LK, Arnold SV, Bittner V, Brewer LC, Demeter SH, et al. 2023 AHA/ACC/ACCP/ASPC/NLA/PCNA Guideline for the Management of Patients With Chronic Coronary Disease: A Report of the American Heart Association/American College of Cardiology Joint Committee on Clinical Practice Guidelines. Circulation. 2023;148(9):e9-e119.

5. Gulati M, Levy PD, Mukherjee D, Amsterdam E, Bhatt DL, Birtcher KK, et al. 2021 AHA/ACC/ASE/CHEST/SAEM/SCCT/SCMR Guideline for the Evaluation and Diagnosis of Chest Pain: A Report of the American College of Cardiology/American Heart Association Joint Committee on Clinical Practice Guidelines. Circulation. 2021;144(22):e368-e454.

6. Genders TS, Steyerberg EW, Alkadhi H, Leschka S, Desbiolles L, Nieman K, et al. A clinical prediction rule for the diagnosis of coronary artery disease: validation, updating, and extension. Eur Heart J. 2011;32(11):1316-30.

7. Pryor DB, Shaw L, McCants CB, Lee KL, Mark DB, Harrell FE, Jr., et al. Value of the history and physical in identifying patients at increased risk for coronary artery disease. Ann Intern Med. 1993;118(2):81-90.

8. Genders TS, Steyerberg EW, Hunink MG, Nieman K, Galema TW, Mollet NR, et al. Prediction model to estimate presence of coronary artery disease: retrospective pooled analysis of existing cohorts. BMJ. 2012;344:e3485.

9. Winther S, Murphy T, Schmidt SE, Bax JJ, Wijns W, Knuuti J, et al. Performance of the American Heart Association/American College of Cardiology Guideline-Recommended Pretest Probability Model for the Diagnosis of Obstructive Coronary Artery Disease. J Am Heart Assoc. 2022;11(24):e027260.

10. Juarez-Orozco LE, Saraste A, Capodanno D, Prescott E, Ballo H, Bax JJ, et al. Impact of a decreasing pre-test probability on the performance of diagnostic tests for coronary artery disease. Eur Heart J Cardiovasc Imaging. 2019;20(11):1198-207.

11. Garcia M, Mulvagh SL, Merz CN, Buring JE, Manson JE. Cardiovascular Disease in Women: Clinical Perspectives. Circ Res. 2016;118(8):1273-93.

12. Hemal K, Pagidipati NJ, Coles A, Dolor RJ, Mark DB, Pellikka PA, et al. Sex Differences in Demographics, Risk Factors, Presentation, and Noninvasive Testing in Stable Outpatients With Suspected Coronary Artery Disease: Insights From the PROMISE Trial. JACC Cardiovasc Imaging. 2016;9(4):337-46.

13. Graham G. Racial and Ethnic Differences in Acute Coronary Syndrome and Myocardial Infarction Within the United States: From Demographics to Outcomes. Clin Cardiol. 2016;39(5):299-306.

14. DeVon HA, Burke LA, Nelson H, Zerwic JJ, Riley B. Disparities in patients presenting to the emergency department with potential acute coronary syndrome: it matters if you are Black or White. Heart Lung. 2014;43(4):270-7.

15. Lopez L, Wilper AP, Cervantes MC, Betancourt JR, Green AR. Racial and sex differences in emergency department triage assessment and test ordering for chest pain, 1997-2006. Acad Emerg Med. 2010;17(8):801-8.

16. Zaman MJ, Junghans C, Sekhri N, Chen R, Feder GS, Timmis AD, et al. Presentation of stable angina pectoris among women and South Asian people. CMAJ. 2008;179(7):659-67.

17. King-Shier K, Quan H, Kapral MK, Tsuyuki R, An L, Banerjee S, et al. Acute coronary syndromes presentations and care outcomes in white, South Asian and Chinese patients: a cohort study. BMJ Open. 2019;9(3):e022479.

18. Armoundas AA, Narayan SM, Arnett DK, Spector-Bagdady K, Bennett DA, Celi LA, et al. Use of Artificial Intelligence in Improving Outcomes in Heart Disease: A Scientific Statement From the American Heart Association. Circulation. 2024;149(14):e1028-e50.

19. Al-Zaiti S, Besomi L, Bouzid Z, Faramand Z, Frisch S, Martin-Gill C, et al. Machine learning-based prediction of acute coronary syndrome using only the pre-hospital 12-lead electrocardiogram. Nat Commun. 2020;11(1):3966.

20. Han C, Kang KW, Kim TY, Uhm JS, Park JW, Jung IH, et al. Artificial Intelligence-Enabled ECG Algorithm for the Prediction of Coronary Artery Calcification. Front Cardiovasc Med. 2022;9:849223.

21. Tang P, Wang Q, Ouyang H, Yang S, Hua P. The feasibility of early detecting coronary artery disease using deep learning-based algorithm based on electrocardiography. Aging (Albany NY). 2023;15(9):3524-37.

22. Yuan N, Duffy G, Dhruva SS, Oesterle A, Pellegrini CN, Theurer J, et al. Deep Learning of Electrocardiograms in Sinus Rhythm From US Veterans to Predict Atrial Fibrillation. JAMA Cardiol. 2023;8(12):1131-9.

23. Hughes JW, Tooley J, Torres Soto J, Ostropolets A, Poterucha T, Christensen MK, et al. A deep learning-based electrocardiogram risk score for long term cardiovascular death and disease. NPJ Digit Med. 2023;6(1):169.

24. Winther S, Schmidt SE, Rasmussen LD, Juarez Orozco LE, Steffensen FH, Botker HE, et al. Validation of the European Society of Cardiology pre-test probability model for obstructive coronary artery disease. Eur Heart J. 2021;42(14):1401-11.

25. Lee UW, Ahn S, Shin YS, Kim YJ, Ryoo SM, Sohn CH, et al. Comparison of the CAD consortium and updated Diamond-Forrester scores for predicting obstructive coronary artery disease. Am J Emerg Med. 2021;43:200-4.

26. Baskaran L, Danad I, Gransar H, B OH, Schulman-Marcus J, Lin FY, et al. A Comparison of the Updated Diamond-Forrester, CAD Consortium, and CONFIRM History-Based Risk Scores for Predicting Obstructive Coronary Artery Disease in Patients With Stable Chest Pain: The SCOT-HEART Coronary CTA Cohort. JACC Cardiovasc Imaging. 2019;12(7 Pt 2):1392-400.

27. Birnbaum Y, Wilson JM, Fiol M, de Luna AB, Eskola M, Nikus K. ECG diagnosis and classification of acute coronary syndromes. Ann Noninvasive Electrocardiol. 2014;19(1):4-14.

28. Al-Zaiti S, Macleod R, Dam PV, Smith SW, Birnbaum Y. Emerging ECG methods for acute coronary syndrome detection: Recommendations & future opportunities. J Electrocardiol. 2022;74:65-72.

29. Al-Zaiti SS, Martin-Gill C, Zegre-Hemsey JK, Bouzid Z, Faramand Z, Alrawashdeh MO, et al. Machine learning for ECG diagnosis and risk stratification of occlusion myocardial infarction. Nat Med. 2023;29(7):1804-13.

30. Sangha V, Nargesi AA, Dhingra LS, Khunte A, Mortazavi BJ, Ribeiro AH, et al. Detection of Left Ventricular Systolic Dysfunction From Electrocardiographic Images. Circulation. 2023;148(9):765-77.

31. Ramirez-Moreno DF, Schwartz O, Ramirez-Villegas JF. A saliency-based bottom-up visual attention model for dynamic scenes analysis. Biol Cybern. 2013;107(2):141-60.

32. Hicks SA, Isaksen JL, Thambawita V, Ghouse J, Ahlberg G, Linneberg A, et al. Explaining deep neural networks for knowledge discovery in electrocardiogram analysis. Sci Rep. 2021;11(1):10949.
